# Rheological, thermal, and microstructural data of lemon essential oil structured with fatty gelators

**DOI:** 10.1016/j.dib.2020.106014

**Published:** 2020-07-12

**Authors:** Sergio Cabrera, John Rojas

**Affiliations:** College of Pharmaceutical and Food Sciences, University of Antioquia, Street 67 # 53-108, Medellín, Colombia

**Keywords:** Citrus lemon essential oil, Crystal structure, Gelators, Lipidic matrices, Rheology

## Abstract

Traditionally, oil structuring has been conducted merely in vegetable oils. Alternatively, essential oil structuring provides a great opportunity to develop the topical application of these oils without causing allergic contact dermatitis and improving the sensory properties. The thermal, rheological and microstructure data collection of lipidic matrices produced with representative gelators such as carnauba wax, stearic acid, glyceryl monostearate and hydroxyethyl cellulose in lemon essential oil were carried out by DSC, rheology and phase contrast microscopy measurements. This dataset is valuable to researchers interested in characterizing lipidic matrices produced with several gelators, once incorporated in different topical formulations. These data can be used for quality control of topical formulations having several textural features.

Specifications table

**Table:** 

Subject	Pharmaceutical science
Specific subject area	Pharmaceutical science, biomaterials
Type of data	Table Image Figure
How data were acquired	DSC: 200 PC, Nietzsche, Feinmahltechnik GmbH, Germany Diffractometer: Empyrean 2012, Westborough, MA, USA Rheometer: MCR92, Anton Paar, Graz, Austria Phase contrast microscope: IN300, Amscope, Germany
Data format	Raw Analyzed Filtered
Parameters for data collection	Lipidic matrices were prepared by adding 45 mg of gelator to ∼3 mL of lemon essential oil. The mixture was then heated at 80 °C under mild stirring (200 rpm) using a heating plate coupled with a magnetic stirrer (Gehaka, MS7-H550-S, São Paulo, Brazil) until a clear solution was obtained (5 min). The dispersion thus obtained was allowed to cool down to 25 °C within two minutes before testing.
Description of data collection	The thermal profiles of pure gelators and lipidic matrices were examined with a DSC equipped with a refrigerated cooling system. Nitrogen was used as purge gas. The cell constant and temperature were set with indium. The sample (weighing from 9 to 12 mg/cup) was placed inside an aluminum pan and sealed with an aluminum lid. Samples were equilibrated at 5 °C and then heated to 80 °C (heating step) at a rate of 10 °C/min followed by a cooling cycle to 5 °C (cooling step) at the same rate. Diffractograms were obtained on a PANalytical diffractometer operated at 40 kV, and 30 mA, equipped with a monochromatic CuK_1_ = 1.5460 A°,k_2_ = 1.54438 A° X-ray diffraction. Diffractograms were acquired over a 5–35° 2^Θ^ range and step scan and step time of 0.039 and 32 s, respectively. Rheological measurements were carried out using an advanced rheometer equipped with a Peltier system for temperature control. A parallel plate (cross-hatched; diameter, ϕ = 25.0 mm; gap = 400 µm) geometry was used to determine the linear visco-elastic region of the lipidic matrices. Microphotographs were obtained on a Phase contrast microscope equipped with a CCD-MT camera.
Data source location	Cenqfal Instrumentation Center, University of Antioquia, Medellin, Colombia
Data accessibility	With the article
Related research article	Sergio Cabrera, John Rojas, Ana Naranjo, Gelmy Ciro. 2020. Lipidic matrixes containing lemon essential oil increases storage stability: Rheological, thermal, and microstructural Studies. Applied sciences. 10 (11), 3909, 1–18. https://doi.org/10.3390/app10113909

## Value of the data

Researchers in the pharmaceutical, cosmetic and clinical fields are interested in characterizing lipidic matrices, especially those intended for topical applications.

• The thermal and microscopy data can be used in quality control laboratories.
• The gelling parameters of these lipidic matrices can be used to further examine and predict the shelf-life of other essential oil-based matrices.

## 1 Data description

### 1.1 Crystallization behavior

Table 1 lists the wide angle X-Ray parameters of neat gelators and their lipidic matrices. Except for hydroxyethyl cellulose (HEC), most waxes showed a degree of crystallinity (DC) >35%. The hydroxyethyl moieties introduce a greater disorder and reflections at 12, 20 and 22° 2^Θ^, which are typical of the 11¯0, 110 and 200 reflections of the monoclinic unit cell and provide a loose molecular packing. Stearic acid showed a high DC and showed reflections at 7, 21.4 and 23.7° 2^Θ^ corresponding to the typical reflections of the E allomorph [1]. Carnauba wax (CRW) was the only heterogeneous gelator in which wax ester compounds (WEs) prevailed showing one strong reflection peak at 21.4°, followed in intensity by a second peak at 23.7° The (110) reflection peak corresponds to a triple chain length stacking (3 L), whereas the (200) peak is indicative of a 2 L packing. These results indicate a β′ orthorhombic crystalline sub-cell arrangement [2]. Conversely, glyceryl monostearate (GMS) showed reflections at 19.4° and 23.0° 2^Θ^ attributed to the β-form which is the most stable packing form having the lowest thermodynamic energy [3].

**Table 1:** Thermal and XRD parameters.

Fatty gelator or lipidic matrix	Tm _Onset_ (°C)	Tm (°C)	Tm _offset_ (°C)	Tc _onset_ (°C)	Tc (°C)	Tc _offset_ (°C)	∆T (°C)	ΔHm (J/g)	ΔHc (J/g)	DC (%)	2Θ Angle (°)
Stearic acid	41.7, 52.9	50.1* 57.0	52.9, 64.4	52.2	50.5	39.2	12.2	62.8	16.8	79.3	7.01, 21.4, 23.7
Glyceryl monoestearate	52.1	56.7	64.3	55.2, 52.4	51.9*, 54	52.4, 44.2	9.1	9.7	1.5, 0.6	N.A	19.4, 22.9
Carnauba wax	60.0	82.0	85.0	71.7	61.5	51.7	17.0	13.3	0.4	70.6	21.4, 23.7
Hydroxyethyl cellulose	33.7	51	62.0	73.7	69.8	63.1	11.7	38	1.0	35	12, 20, 22
Stearic acid matrix	17.0, 40.6	24.8 49.1	30.4, 51.7	34.4	31.9	22.5	17.3	1.2, 1.8	1.4	N.A	N.A
Glyceryl monoestearate matrix	39.4	64.1	53.4	51,0	68.6	69,0	2.4	2.3	0.2	N.A	N.A
Carnauba wax matrix	37.0	49.4	54.8	61.0	54.7	47.2	6.2	0.4	0.4	N.A	N.A
Hydroxyethyl cellulose matrix	31.4	64	58.5	63.2	68.3	71.1	4.7	7.1	0.9	N.A	N.A

*A shoulder from the main peak; NA: Not applicable.

**Fig. 1:**
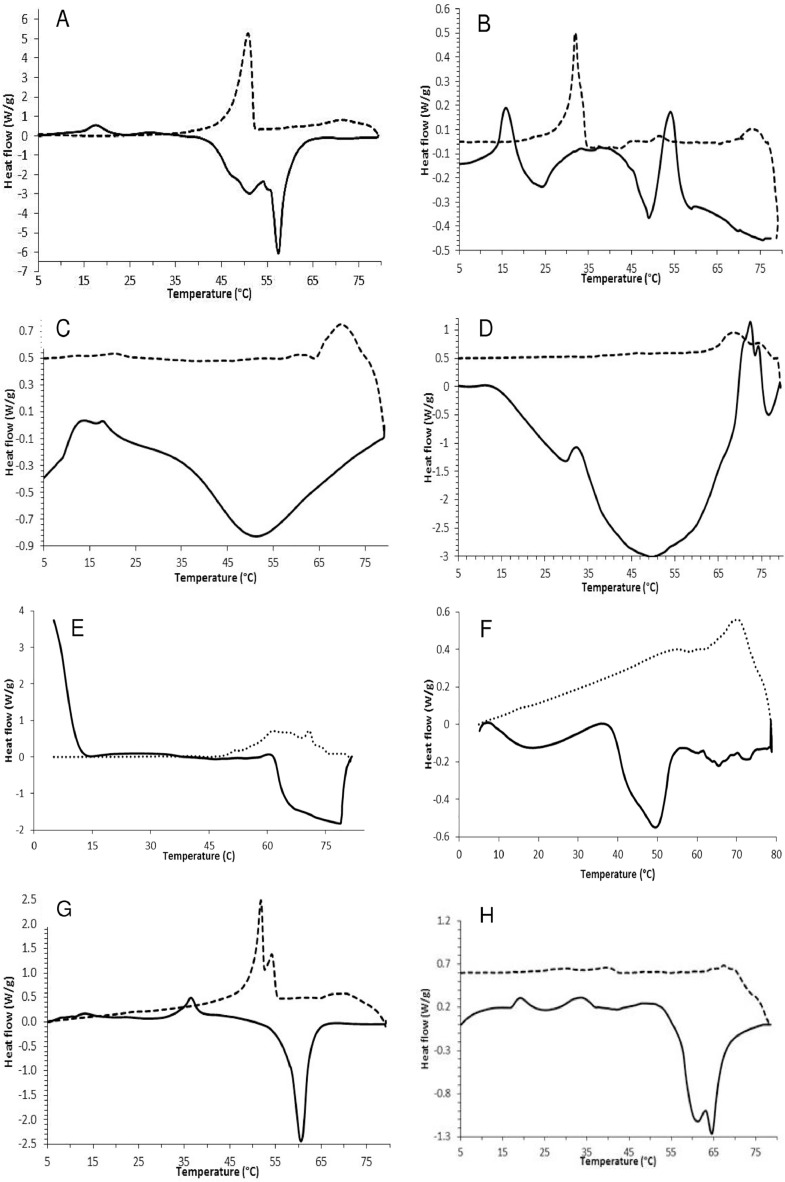
Differential scanning calorimetric profiles of: (A) stearic acid, (B) stearic acid-based matrix, (C) hydroxyethyl cellulose, (D) hydroxyethyl cellulose-based matrix, (E) carnauba wax, (F) carnauba wax-based matrix, (G) glyceryl monostearate, (H) glyceryl monostearate-based matrix. Straight and dotted lines correspond to the heating and cooling curves, respectively.

Fig. 1 depicts the thermograms resulted from the crystallization profile (dotted lines) and melting profile (straight lines) of the neat gelators (left panel) and their corresponding lipidic matrices (right panel). The crystallization peaks of neat gelators were smaller in magnitude than the melting peaks. HEC due to its semi-crystalline nature showed one large and wide endothermic event, and this peak did not correspond to the melting event which is known to be between 160 and 220 °C. Interestingly, gelators such as stearic acid (SA) showed a main peak at 57 °C along with a shoulder at 50.1 °C. This could indicate the presence of impurities resulted from the purification process from the shea or cocoa butter source. Usually, commercial SA contains a mixture of lauric acid and palmitic acid up to a 25% which are responsible for the wide and low melting point. A shoulder at ∼50 °C is also indicative of this heterogeneous character. Likewise, GMS showed only one endothermic event at 56.7 °C, which is much lower than that reported at 72 °C. This phenomenon is explained by the partial esterification of the material. The large exothermic band with a large shoulder depicts partial co-crystallization at 51–54 °C due to the residual diester fraction.

**Fig. 2:**
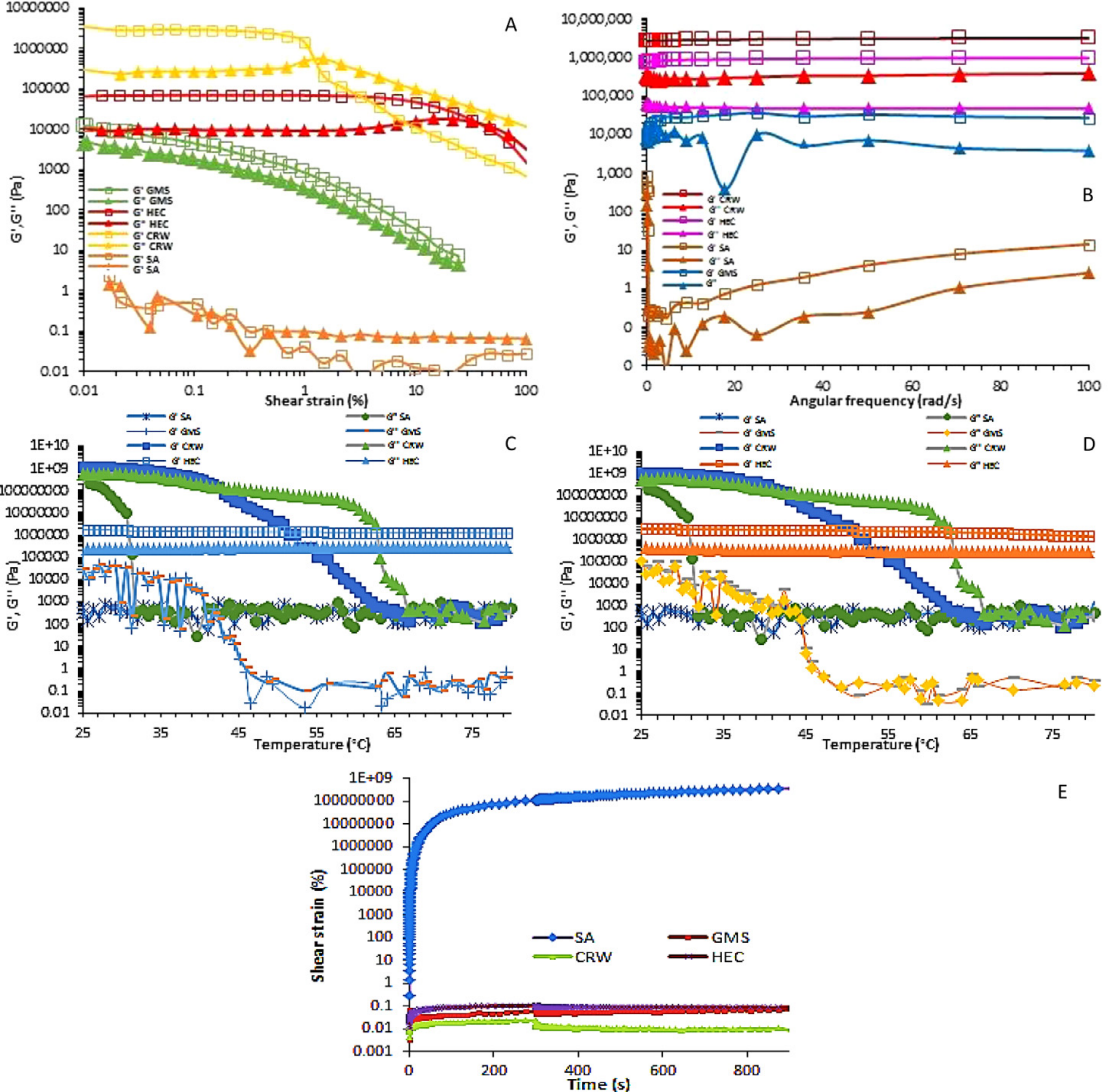
Linear viscoelastic region (A); frequency sweep curves (B); heating temperature sweep (c) cooling temperature sweep (d) creep test (e) of the matrices produced with HEC, hydroxyethyl cellulose; CRW, carnauba wax; SA, stearic acid; GMS, glyceryl monostearate.

The right panel in Fig. 2 shows the thermograms resulted from the lipidic matrices. They are shown as amorphous systems due to the very small crystallization peaks. The presence of LEO broadened the melting point of these matrices. Further, SA-based matrices showed two endothermic events, but only one crystallization event. It also showed the lowest melting temperature indicating a large miscibilization rather than gelation with lemon essential oil (LEO). GMS-based matrices showed a dual endothermic peak between 60 and 65 °C in which the wax ester characteristics prevails. On the other hand, the high amorphization of the lipidic matrices is supported by the energy required to crystallize or melt these systems which was smaller than that of the gelator counterpart. For instance, the crystallization energy of HEC-based matrix was one-eighth the size of its melting energy. This is explained by the partial level of HEC swelling in LEO which took place at room temperature. Conversely, CRW-based matrices showed comparable melting and crystallization energies favoring a crystal formation.

The difference in temperature (∆T) between Tm_offset_-Tc_onset,_ largely depended on the kind of gelator employed. The neat GMS and its corresponding lipidic matrix showed the lowest ∆T and a rapid crystal formation. Conversely, SA-based matrices had the largest ∆T values and a slower network formation as compared to other systems. In this case, the acid moiety might hinder the cohesive forces for interaction with the oil compounds.

### 1.2 Isothermal oscillatory rheological behavior

Fig. 2a and Table 2 show that except for SA-based matrices G′ was higher than G′’ (>50 Pa) within the linear viscoelastic region (LVR) indicating a gel property, and HEC-based matrices showed the largest region, whereas CRW-based matrices showed the largest critical point. These results are translated in a large ductility for HEC matrices, and excellent structural resistance to the applied strain for CRW matrices. The latter matrix showed the highest G’ at cross-over point confirming the strong and a stable network. Further, the CRW matrix was considered as the only strong gel since G’’/G’ was always lower than 0.1, demonstrating that the solid-like properties strongly dominate over the liquid-like properties [4]. Conversely, SA and GMS matrices showed a low and a narrow yield zone indicating a rapid structure breakdown and their lipidic matrices were very soft and yielded at very low strains.

**Table 2:** Viscoelastic parameters obtained from the linear viscoelastic region of oscillation yield strain, frequency and temperature sweep tests. Herschel-Buckley parameters and thixotropic recovery from the stress-strain profiles at 25 °C.

Gelator-based matrix	Critical point _LVR_ (%)	Critical stress (Pa)	Dynamic yield stress (Pa)	Average G’’/G’_LVR_	Temperature sweep		Herschel-Buckley parameter				Recovery (%)
					T_G-S_	T_S-G_	τ_o_ (Pa)	k (Pa.s)	n	r^2^	
Glyceryl monostearate	0.006	187,710	20,588	0.36	45.9	44.4	3.94	1.32	0.75	0.9232	64.5
Hydroxyethyl cellulose	4.67	62,094	11,859	0.17	N.A	N.A	184.9	0.0082	2.13	0.9700	95.7
Stearic acid	0.02	0.53	0.097	0.61	25.0	31.2	0	0.0015	0.97	0.9984	99.9
Carnauba wax	0.32	2,676,100	557,040	0.096	64.8	65.0	72.7	11.19	0.491	0.8200	9.6

T_G-S_: gel-sol transition, T_S-G_: sol-gel transition; n: flow type or flow behavior index, k: consistency index, τ_o_: shear stress for flow.

Most lipidic matrices showed a slight dependence on the frequency from 0.1 to 100 rad/s as indicated by a slightly positive slope in the G′ and G″ curves, especially for SA and GMS matrices which corroborate their soft nature (Fig. 2b). The almost horizontal profile of CRW and HEC-based oleogels indicates an almost parallel trend with the x-axis indicating long-term stability at resting conditions.

### 1.3 Gelling behavior

Lipidic matrices exhibited higher values of G’ than those of G’’ at low temperatures, showing a dominant elastic property (solid-like state). During the heating phase G′ and G″ values showed a more pronounced decrease for CRW and GMS-based matrices, whereas HEC and SA-based matrices showed almost no decrease with increasing temperature (Fig 2c). A plateau followed by one abrupt transition is observed within the range between 55 and 65 °C for CRW-based matrices and corresponds to the T_G-S_ phase transition. Once the crossover point is surpassed, the curves are very alike which is explained by the complete melting of the gelator network within the matrix. Interestingly, during the whole heating stage HEC-based matrices remained as a gel phase whereas, SA-based matrices remained as a sol phase.

The poor solubilization of SA within the LEO became more prominent during the cooling phase. Thus, below the melting point of the gelator phase separation occurs, resulting in an incomplete gelation (Fig 2d). Further, the T_S-G_ of SA-based matrices matched that of T_c_ (31.2 °C). This indicates that these two processes occurred almost simultaneously or one event is a consequence of the other. Further, matrices formed by CRW, and HEC crystals achieved a strong gelling behavior (G’ -G’’ ≥ 1 decade), whereas SA-based matrices behaved as a weak gel (G’–G’’ < 1 decade).

### 1.4 Isothermal flow and thixotropic behavior

The creep test was conducted to assess the recovery ability of the matrices (Fig. 2e). Thus, by applying a constant stress of 50 Pa for 25 s lipidic matrices deformed, but once that stress ceased, HEC and CRW-based matrices suffered from an abrupt fall in deformation showing some tendency to recovery (Table 2). However, SA and GMS showed minimal recovery and continued flowing. Thus, as the system is deformed, with time, the structure breaks down into smaller clusters of aggregates flowing, and the restructuring of these clusters into a coherent network is avoided because the Brownian motion is overcome by the shear force [5].

The flow profiles of these matrices were fitted to the Herschel-Buckley model and results are listed in Table 2. Except for the SA-based matrix which showed a truly Newtonian behavior, CRW and GMS-based matrices showed a pseudoplastic character or shear thinning flow behavior (*n* < 1). Interestingly, the HEC-based matrix showed a shear thickening character. The K parameter measures the thickness of the fluid which is very close to the viscosity at very low shear rates and CRW-based matrix showed the largest value. In fact, the CRW-based matrix also exhibited the largest degree of shear thinning. As expected the HEC-based matrix presented the largest yield stress for flow (t_o_) due to its shear thickening character. Conversely, the SA-based matrix was very weak in strength, and showed an absence in yield stress indicating immediate flow under applied shear.

### 1.5 Crystal morphology

**Fig. 3:**
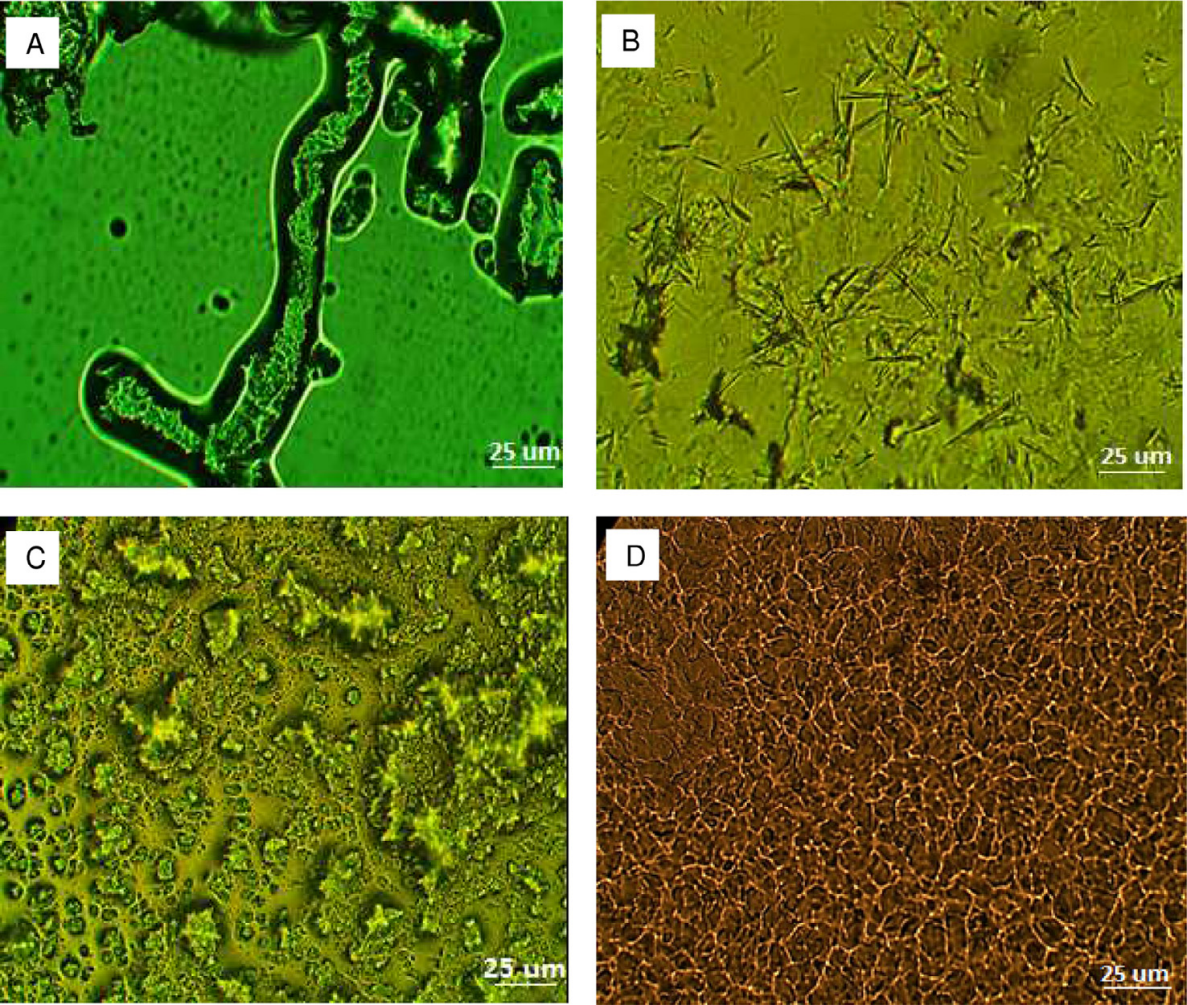
Phase contrast microphotographs of lipidic matrices of lemon essential oil prepared with Hydroxyethyl cellulose (A), stearic acid (B), carnauba wax (C) and glyceryl monostearate (D).

Fig. 3 depicts the morphological features of the matrices as seen by the phase contrast microphotographs. HEC matrices showed the largest fiber-like structure or strands which absorbed the oil and led to partial swelling. Thus, with the applied shear stress these fibers are able to intertwine resulting in large values of G’ and G’’. On the other hand, SA-based matrices crystallized into very fine needles imbrating together which are further organized into an open aggregate-like structure. These needles were very thin, elongated and fragile having from 20 to 30 µm in length. CRW-based matrices had an anisotropic, grain-like morphology with lengths of 5–10 µm range. GMS-based matrices developed mostly small spherulitic and grainy crystals rather that fibers of 5–10 µm in size. They appeared to be highly densified and interconnected to each other.

## 2 Experimental design, materials and methods

### 2.1 Chemicals

Lemon essential oil was purchase from Green Andina (Bogota, Columbia). Gelators were obtained from protokimica (Medellín, Columbia).

### 2.2 Preparation of lipidic matrices

They were prepared by adding 45 mg of gelator to ∼3 mL of lemon essential oil. Samples were heated at 80 °C under mild stirring (200 rpm) using a heating plate coupled with a magnetic stirrer (Gehaka, MS7-H550-S, São Paulo, Brazil) until clear solutions were obtained (5 min). These dispersions were then allowed to cool down to 25 °C within two minutes before testing.

### 2.3 Thermal behavior

The thermal profiles of gelators and lipidic matrices were examined with a DSC (200 PC, Nietzsche, Feinmahltechnik GmbH, Germany) equipped with a refrigerated cooling system. Nitrogen was used as purge gas. The cell constant and temperature were set with indium. The sample (weighing from 9 to 12 mg/cup) was placed inside an aluminum pan and sealed with an aluminum lid. Samples were equilibrated at 5 °C and then heated to 80 °C (heating step) at a rate of 10 °C/min followed by a cooling cycle to 5 °C (cooling step) at a rate of 10 °C/min. Characteristic parameters of the thermal curves, including onset temperature (Tc_onset_ and Tm_onset_), peak maximum temperature (Tc and Tm), offset temperature (Tc_offset_ and Tm_offset_), melting and crystallization enthalpies (ΔHm and ΔHc) and ∆T (Tc_onset_ -Tm_offset_) were obtained using the Nietzsche Analysis software.

### 2.4 Phase contrast microscopy

The microstructure of wax crystals of the lipidic matrices was observed using an inverted phase contrast microscope (IN300, Amscope, Germany) equipped with a color camera CCD-MT (Amscope Vs. 3.7, Corp, USA) at a 400X magnification.

### 2.5 X-ray diffraction

Polymorphism of the wax crystals was investigated on a PANalytical diffractometer (Empyrean 2012, Westborough, MA) operated at 40 kV, and 30 mA, equipped with a monochromatic Cu Kα_1_ = 1.5460 A°,kα_2_ = 1.54438 A° X-ray diffraction. Diffractograms were obtained over a 5–35° 2^Θ^ range and step scan and step time of 0.039 and 32 s, respectively. The degree of crystallinity was calculated using the Peak fit software (Seasolve^Ⓡ^, Inc Framingham, MA).

### 2.6 Rheological behavior

Rheological measurements were carried out using an advanced rheometer (MCR92, Anton Paar, Graz, Austria) equipped with a Peltier system for temperature control. A parallel plate (cross-hatched; diameter, ϕ = 25.0 mm; gap = 400 µm) geometry was used to determine the linear visco-elastic region of the matrices

#### 2.6.1 Isothermal measurements (Strain and frequency sweeps)

Strain sweeps at a constant frequency of 1 Hz were performed to determine the linear viscoelastic region (LVR) of matrices. The strain ramp was conducted between 0.01 to 100%, at 1 Pa, and 25 °C. The time-dependent deformation behavior of matrices was conducted at angular frequency ranging from 0.1 to 100 rads^−1^ and 1 Pa with a strain value within the LVR (0.01%).

#### 2.6.2 Temperature sweeps (non-isothermal measurements)

Temperature ramps were performed simulating the gel network formation with a fixed frequency (1 Hz), strain (0.01%) and stress value (1 Pa) set within LVR. Initially, the equipment was maintained at 25 °C followed by a heating step to 80 °C, maintained isothermally for 5 min, followed by a cooling step to 25 °C.

#### 2.6.3 Thixotropy (isothermal measurement)

The capacity of lipidic matrices to recover viscosity after shear was evaluated by means of thixotropic recovery tests. First, samples were subjected to two consecutive cycles of shear rates from 0.1 t0 100 s^−1^ with step time of 10 s and then maintained at 100 s^−1^ for 5 min followed by decreasing rate to 0.1 *s* ^−^ ^1^.The viscosity recovery percentage of these matrices was calculated by comparing the AUC from the increasing and decreasing shear rate steps. Flow curves were fitted to the general Herschel-Bulkley model with three parameters using the Statgraphics^Ⓡ^ software. Further, the Creep test was conducted at two stages. The first one submitting the samples to a constant stress of 50 Pa for 25 s followed by a stress release for 25 s and measuring the strain change.

## Declaration of Competing Interest

The authors declare that they have no known competing financial interests or personal relationships which have, or could be perceived to have, influenced the work reported in this article.

## Appendix Supplementary materials

Image, application 1

Image, application 2
